# Virulence Potential of Escherichia coli Isolates from a Beef Farm in Puerto Rico

**DOI:** 10.1128/mra.00443-22

**Published:** 2022-10-26

**Authors:** Yadira Malavez, Paola N. Loperena-Gonzalez, Sharon M. Nieves-Miranda, Enilanice Vazquez-Rodriguez, Carlos N. Centeno-Velez, Lingzi Xiaoli, Edward G. Dudley

**Affiliations:** a Department of Natural Sciences, University of Puerto Rico, Aguadilla, Puerto Rico; b Department of Food Science, The Pennsylvania State University, University Park, Pennsylvania, USA; c *E. coli* Reference Center, The Pennsylvania State University, University Park, Pennsylvania, USA; University of Maryland School of Medicine

## Abstract

Sixteen Escherichia coli isolates were obtained from fecal matter from a beef farm in Puerto Rico. Isolates were whole-genome sequenced for *in silico* characterization, including pathotype characterization, virulence, and plasmid identification. The results of the draft genomes identified potential pathogenic E. coli strains from beef cattle in Puerto Rico.

## ANNOUNCEMENT

Shiga toxin-producing E. coli (STEC) strains are foodborne pathogens that cause diarrhea, hemorrhagic colitis, and life-threatening hemolytic uremic syndrome (HUS). Annually, STECs cause approximately 2.8 million illnesses, 3,890 HUS cases, and 230 fatalities, worldwide (1–3). The O157 and non-O157 serotypes caused 2,363 and 3,646 cases in 2017 in the USA, respectively (4).

Beef is an important agricultural commodity of Puerto Rico (PR), with $37.7 million in annual sales (5, 6). The characterization of E. coli strains in beef cattle (<5 years old) and calves (0 to 3 months old) will provide data that can be used to create awareness about their potential pathogenicity for public health analysis.

Thirty-five fecal samples were collected from a beef farm in PR. Samples were collected from the rectum using sterile gloves (cattle) or sterile swabs (calves) and were transported on ice to a laboratory within 2 h (7, 8). The UPR Institutional Animal Care and Use Committee (IACUC) provided ethical clearance under the protocol number 01-2017-IACUC-UPRAg (7, 8). The samples were suspended in 10 mL of 0.09% NaCl (1:10), and 1 mL was transferred to tryptic soy broth (9). *E coli* was identified by culture and by biochemical tests: MacConkey-lactose agar, sulfur-indole-motility, and methyl red/Voges-Proskauer. The media were was incubated aerobically for 24 h at 37°C.

DNA from a single colony was extracted using a Qiagen DNeasy Blood Kit (Qiagen, Hilden, Germany), and 1 mL was extracted from a Luria-Bertani culture for 18 h at 37°C. Separate genomic libraries were created using the Nextera-XT Kit (Illumina, Inc., San Diego, CA) and sequenced using an Illumina MiSeq. The paired end reads (2×, 250 bp) were uploaded to GalaxyTrakr (10). Quality was assessed using FastQC v.0.72 (11). The genomes were *de novo* assembled with SPAdes v.3.12.0, using k-mer lengths of 127 (12). Assembly quality assessment with QUAST v5.0 (13). Skesa MLST v.0.0.3 (14), VirulenceFinder v.2.0 (15), and ECTyper v1.0.0 (16), were used for characterizing the sequence types (ST), virulence, and serotypes, respectively. Phylogroups were identified *in silico* using ClermontPhylotyper v21.03 (17). The STEC pathotype was classified by identifying at least one of the *stx*_1_ and *stx*_2_ genes (18, 19). ResFinder (v4.1) (15), Virulence Finder (v2.0) (20), and PlasmidFinder (2.1) were used to identify the resistance, virulence, and plasmids, respectively (20, 21). Default parameters were used for all software unless specified.

The assembly length ranged from 4,635,908 to 5,549,290 bp (Table 1). The identified pathotypes included seven STECs (cattle: 2/8, calves: 5/8 calves). Four isolates carried the *stx2a* subtype, which was associated with HUS (20). Fifteen ST were detected, including one associated with serotype O157:H7. The intimin gene (*eae*) identified in calves (4/8) is characteristic of virulent isolates. The *eae* gene is encoded within the locus of enterocyte and effacement (LEE) that is responsible for lesions on the intestinal mucosa, along with the secreted effector protein (Esp), translocated intimin receptor (Tir), and other effectors (22). Nine IncF plasmids replicons were identified, contributing to the spread and the plasticity of the virulence genes (23).

**TABLE 1:** *In silico* characterization of 16 E. coli genomes isolated from beef cattle and calves from a farm in Puerto Rico

SRA accession no.	Assembly length (bp)	*N_50_*	GC%	Total reads	Contigs	Sequence type (ST)	Serotype	BioSample	Assembly	Pathotype	Phylogroup	Virulence genes	Plasmids
Cattle												
SRR10990379	5,549,290	73,280	50	643,704	210	2,715	O2:H25	SAMN13945463	GCA_015316345.1	-^*a*^	-^*a*^	*astA, ehxA, espI, iss*	IncFIA, IncFIB(AP001918), IncFII(pHN7A8)
SRR10990345	5,180,466	78,121	51	794,402	226	677	O174:H21	SAMN13945466	GCA_0126463651	STEC	B1	*iha, iss, ipfA, stx2a, stx2b*	IncF1A, IncF2B, (AP001918) IncFII(pHN7A8)
SRR10990325	4,995,688	38,313	51	621,725	297	2,485	O15:H45	SAMN13945073	GCA_012646145.1	-^*a*^	D	*Iss*	IncF2B(AP001918), IncFIA
SRR10990233	5,064,538	375,813	51	431,255	45	998	O2:H6	SAMN13945411	GCA_012669965.1	-^*a*^	B2	*iss, kpsMTII, papC, sfaS, vat*	-^*a*^
SRR10987151	5,022,203	107,170	51	729,469	128	2,485	O15:H45	SAMN13941749	GCA_012648475.1	-^*a*^	D	*iss, sfaS*	IncF2B(AP001918), IncFIA
SRR10990259	4,635,908	224,014	51	572,086	63	2,144	O81:H14	SAMN13945423	GCA_012646525.1	-^*a*^	B1	*ipfA, iss*	-^*a*^
SRR10990254	315,829	100,259	50	719,128	177	6,475	O73:H45	SAMN13945456	GCA_015316385.1	STEC	A	*ehxA, espI, espP, iha, iss, stx1a, stx1b, stx2a, stx2b*	IncFIB(AP001918)
SRR10992555	4,963,407	58,215	51	503,376	200	297	O169:H8	SAMN13945468	GCA_012593855.1	-^*a*^	B1	*ipfA*	IncFIB(AP001918), IncFII(29), IncI1-I(Alpha)
Calves												
SRR10990263	5,276,923	38,787	51	440,070	459	16	111:H8	SAMN13945061	GCA_016575655.1	STEC	B1	*astA, eae, ehxA, espA, espF, espJ, ipfA, iss, stx1a, stx1b, tccP, tir*	IncFII(pRSB107), IncFII, Col156
SRR10990330	4,694,938	60,647	51	548,215	196	2,728	O113:H8	SAMN13945070	GCA_012658015.1	-^*a*^	B1	*ipfA, iss*	-^*a*^
SRR10990266	4,833,948	35,171	50	543,087	375	8,264	O49:H16	SAMN13945064	GCA_012662345.1	STEC	A	*astA, eae, espA, espF, espI, espP, iha, stx2a, stx2b, tir*	IncB/O/K/Z, IncFIB(AP001918), Col156
SRR10990329	5,318,624	67,824	50	906,928	302	11	O157:H7	SAMN13945065	GCA_012646305.1	STEC	E	*astA, chu, eae, ehxA, espA, espB, espF, espJ, espP, iha, iss, stx2a, stx2b, tccP, tir*	IncFII; IncFIB(AP001918)
SRR10987147	5,420,692	34,358	51	806,222	511	17	O118:H2	SAMN13941735	GCA_012648725.1	STEC	B1	*eae, ehxA, espA, espB, espF, espJ, espP, iss, stx1a, stx1b, tccp, tir*	IncB/O/K/Z, IncFIB(AP001918)
SRR10990225	5,073,410	86,916	51	599,961	206	906	O74:H8	SAMN13945062	GCA_012647805.1	-^*a*^	B1	*ehxA, ipfA, espP, iha, iss*	IncB/O/K/Z, IncFIB(AP001918)
SRR10987129	5,123,926	106,856	51	917,486	237	7,715	O130:H38	SAMN13941700	GCA_012648865.1	STEC	B1	*iha, ipfA, iss, stx1a, stx1b, sxt2a, stx2b*	IncFII(pHN7A8)
SRR10987122	4,702,352	71,246	51	745,178	168	2,328	O54:H2	SAMN13941687	GCA_012648825.1	-^*a*^	B1	*ipfA, iss*	p0111

a -, Not found.

The results suggest that beef calves in Puerto Rico can be a source of STECs. To our knowledge, we provide the first baseline characterization of E. coli potential virulence from beef cattle in PR.

### Data availability.

The sequence reads and assembled genomes were deposited in NCBI under BioProject number PRJNA357722. The SRA accession numbers are SRR10990379, SRR10990345, SRR10990325, SRR10990233, SRR10987151, SRR10990259, SRR10990254, SRR10992555, SRR10990263, SRR10990330, SRR10990266, SRR10990329, SRR10987147, SRR10990225, SRR10987129, and SRR10987122.
